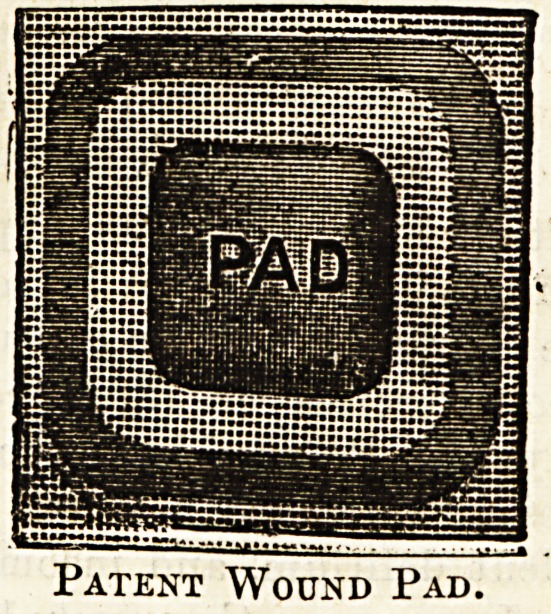# New Appliances and Things Medical

**Published:** 1894-03-24

**Authors:** 


### NEW APPLIANCES AND THINGS MEDICAL.

[All preparations, appliances, novelties, &c., of which a notice is desired, should be sent for the Editor, to care of The Manager, 428, Strand, London, W.C.]

### BURROUGHS AND WELLCOME'S OPHTHALMIC "TABLOIDS" AND CHROMATIC DIAL.

We have received from Messrs. Burroughs and Wellcome the above-mentioned articles, which fairly enough represent the tendency of the modern manufacturer to enter upon undertakings which lie entirely beyond his natural province. The "ophthalmic 'tabloids'" are sent to us in a case which contains nine little bottles of medicated wafers, two camel's hair pencils, a tiny pestle and mortar, a dropping tube, and a "tabloidholder," by means of which some of the wafers are to be applied to the eye of the patient, while others are to be dissolved in water by the aid of the pestle and mortar, so as to make a lotion which might be prepared by any dispensing chemist. The case is accompanied by a printed paper, in which medical practitioners are solemnly informed that the "methods generally in use for the treatment of diseases of the eye are far from being satisfactory." Moreover, "the frequent instillation of a solution is often very distressing and irritating," and other evil consequences of the methods "generally in use" are described. But there is balm in Gilead; for Messrs. Burroughs and Wellcome are not only prepared to teach ophthalmic surgeons how to treat their patients, but to teach them also how applications to the eye should be made. This is an age of progress; and, with the assistance of Messrs. Burroughs and Wellcome and their congeners, it will soon be possible to dispense with a costly medical education, and to make a very good surgeon, ophthalmic or other, mechanically, out of a piece of tin and a watchspring. The individual requirements of the patient will cease to be of importance, the art of prescribing will be regarded as a survival from bygone ages, and the practitioner will find in one or other of his pockets a case of bottles warranted to relieve him of all responsibility. The sole remaining danger will depend upon his liability to take out the wrong case, to substitute, say, the obstetric for the ophthalmic one, or *vice versâ*. But, as we know too well, accident cannot be absolutely excluded from human affairs.

We have some curiosity to know why Messrs. Burroughs and Wellcome call their little wafers "tabloids." Apart from the horror of a jumble of Greek and Latin into one word, the wafers are not in the least "like" any "tables" that we ever remember to have seen; and, if the English word "wafers" be not good enough, the proper one would manifestly be that used in the pharmacopœia, namely, "Lamellæ." The wafers are said to be prepared with a "perfectly sterile, innocuous, and non-irritating basis," but it is obvious that no surgeon would be justified in taking this statement for granted, and that the composition of the basis ought to be disclosed. It would be of real advantage to obtain cocaine wafers with a basis which would render washing of the eye before an operation unnecessary. As far as we have tested the wafers in the case, they seem to be effective and unirritating, but to have the disadvantage of being more brittle than those prepared in the ordinary way. The "tabloidholder," a word into which a third language enters, is a grotesque absurdity; for the best method of applying wafers to the eye is by a pair of tiny forceps, about an inch and a quarter long, by which the wafer may be picked out of its bottle and placed in the exact position on the eye or eyelid lining that is desired. Taking the case as a whole, the best we can say of it is that it reminds us of the faint praise bestowed by a rich uncle upon the patent corkscrew which had been presented to him by a nephew with "expectations." The recipient pronounced the novelty to be "not much more trouble than the old one."

The "chromatic dial" is intended to be used for testing colour-vision, and it belongs to an order of useless playthings of like kind, the number of which is legion. Any of these playthings will detect colour-blindness, if sufficient time and trouble be devoted to the investigation; but, if despatch and certainty are aimed at, and especially if many people have to be examined, the only trustworthy method is by the use of Holmgren's wools in competent and practised hands. The playthings were in existence before Holmgren took up the question, and they have been used and abandoned by experts in every civilised country. This applies chiefly to congenital colour-blindness, but the instructions sent with the "dial" say that colour-blindness may be acquired, and leave it to be supposed that the "dial" may be used for the detection of this form also. Acquired colour-blindness is usually, or at least often, limited to the centre of the visual field, and sometimes cannot be detected even by Holmgren's wools, because a skein furnishes an intra-ocular image of sufficient size to transcend the boundaries of the affected retinal area. The "dial" is directed to be held in the hand of the person examined, or at least to be within his reach, so that he can himself rotate the movable disc which it contains. Before its colour images would be limited to the yellow spot region, so as to discover the existence of a central defect, it would have to be removed to a distance of about eighty feet. The "dial" seems to us to be troublesome and complicated as applied to the detection of congenital colour-blindness, and to be altogether untrustworthy with reference to that which is acquired. On the reverse of the card of instructions we find a very confusing and unpractical test object for the discovery of astigmatism, with no instructions as to the distance at which it should be placed, and a small series of Snellen's test-types, into which letters which are more difficult than others at the stated distances, and which are omitted from the best sheets, have been introduced. On the cover of the case is a sort of table, purporting to show the strength of the glasses required for presbyopia at successive ages. Of this, it is perhaps sufficient to say that it differs somewhat widely from the similar table which was given by Professor Donders, and, moreover, that such a table, even if correct, is calculated to confirm the ignorant in the common belief that the selection of glasses is a matter of mechanics, capable of being safely accomplished by a "penny-in-the-slot" machine.

### "THE LEICESTER" PATENT WOUND AND BED SORE PADS.

(A. De St. Dalmas and Co., Leicester.)

We have been supplied with a sample box of these new contrivances. They are made in various sizes, from 2 in. by 2 in. to 4 in. by 4 in., prepared with a pad of absorbent wool in the middle, and a self adhesive border round the edge. The ease with which they can be applied will enhance their value to those ignorant of bandaging. If the wool pad had

Patent Wound Pad.

been arranged to the form of a protecting ring, after the manner of the well-known corn plaster, so as to relieve threatening bed sores from all pressure, in a great many cases they would be more valuable than in the present form. But in cases where pressure is not a dangerous element—that is to say, in the ordinary form of healthy wound, we consider them to be a contrivance of considerable merit.

## Figures and Tables

**Figure f1:**